# Parathyroid Tumors: Molecular Signatures

**DOI:** 10.3390/ijms222011206

**Published:** 2021-10-18

**Authors:** Francesca Marini, Francesca Giusti, Teresa Iantomasi, Maria Luisa Brandi

**Affiliations:** 1 Department of Experimental and Clinical Biomedical Sciences, University of Florence, 50139 Florence, Italy; francesca.marini@unifi.it (F.M.); francesca.giusti@unifi.it (F.G.); teresa.iantomasi@unifi.it (T.I.); 2 F.I.R.M.O. Fondazione Italiana Ricerca sulle Malattie dell’Osso (Italian Foundation for the Research on Bone Diseases), 50141 Florence, Italy

**Keywords:** parathyroid tumors, parathyroid adenomas, parathyroid carcinomas, gene mutations, epigenetic signatures

## Abstract

Parathyroid tumors are rare endocrine neoplasms affecting 0.1–0.3% of the general population, including benign parathyroid adenomas (PAs; about 98% of cases), intermediate atypical parathyroid adenomas (aPAs; 1.2–1.3% of cases) and malignant metastatic parathyroid carcinomas (PCs; less than 1% of cases). These tumors are characterized by a variable spectrum of clinical phenotypes and an elevated cellular, histological and molecular heterogeneity that make it difficult to pre-operatively distinguish PAs, aPAs and PCs. Thorough knowledge of genetic, epigenetic, and molecular signatures, which characterize different parathyroid tumor subtypes and drive different tumorigeneses, is a key step to identify potential diagnostic biomarkers able to distinguish among different parathyroid neoplastic types, as well as provide novel therapeutic targets and strategies for these rare neoplasms, which are still a clinical and therapeutic challenge. Here, we review the current knowledge on gene mutations and epigenetic changes that have been associated with the development of different clinical types of parathyroid tumors, both in familial and sporadic forms of these endocrine neoplasms.

## 1. Parathyroid Tumors

The parathyroids are four small endocrine glands located in the neck behind the thyroid. They are the “endocrine controllers” of calcium homeostasis that continuously monitor and regulate serum calcium levels through the synthesis and release of parathyroid hormone (PTH). Primary hyperparathyroidism (PHPT), due to a persistent PTH hypersecretion independent from serum calcium levels, is a pathological idiopathic condition indicative of the presence of hyperactive/hypercellular gland(s) (parathyroid hyperplasia) or parathyroid tumors. PHPT is caused by multiple hyperplastic parathyroids in about 15% of cases, and by parathyroid tumors in approximately 85% of cases [1]. Parathyroid tumors are rare endocrine neoplasms affecting 0.1–0.3% of the general population [2], comprising slow-growing benign PTH-secreting adenomas in almost 100% of cases, atypical parathyroid adenomas in about 1.2–1.3% of cases, and extremely rare malignant carcinoma in less than 1% of cases [3]. Conversely to parathyroid adenomas (PAs), parathyroid carcinomas (PCs) show signs of local invasion and/or distant metastases and are characterized by hyperproduction of massive amounts of PTH (up to about 100-fold higher than that of adenomas) and a severe, commonly untreatable, hypercalcemia that accounts for death in a majority of cases. Atypical parathyroid adenomas (aPAs) are a group of an intermediate form of parathyroid cancer, characterized by specific atypical histological features (i.e., solid growth pattern, fibrous bands, and cellular atypia) and with an uncertain malignant potential, which differs from malignant PCs mainly because of the absence of evident signs of local invasion and metastases [2].

Parathyroid tumors, and the related PHPT, manifest primarily as a sporadic single-gland disease in over 90% of cases, while only about 1 in 10 cases are hereditary familial forms, which can affect from 1 to 4 parathyroids [4] (Table 1). Inherited forms include both familial isolated parathyroid tumors and four autosomal dominant syndromic forms, in which the parathyroid neoplasms are associated with other endocrine and non-endocrine abnormalities.

Parathyroid tumors present a great heterogeneity in their genetic background and molecular features, for both inherited and sporadic forms. Unfortunately, no specific histological characteristics allow pre-operative distinction among PAs, aPAs, and PCs. It is therefore of vital importance to increase our knowledge of genetic, epigenetic, and molecular signatures, which characterize different parathyroid tumor subtypes and drive different tumorigeneses, to identify potential diagnostic biomarkers able to distinguish among different parathyroid neoplastic types, as well as to provide novel therapeutic targets and strategies for these rare neoplasms, which are still a clinical and therapeutic challenge.

## 2. Inherited Isolated Hyperparathyroidism

Familial isolated hyperparathyroidism (FIHP) is a hereditary form of PHPT caused by inherited parathyroid hyperplasia, PAs or PCs, which manifests in at least two members of the same family in absence of association with other diseases or tumors. The disease shows an autosomal dominant pattern of inheritance, but a specific genetic cause for FIPH has not yet been identified, and many affected individuals do not have any identifiable mutation. A small proportion of patients with FIHP-causing parathyroid tumors bear germinal inactivating mutations of the Multiple Endocrine Neoplasia type 1 (*MEN1*) gene or of the Cell Division Cycle 73 (*CDC73*) gene, presumably being incomplete clinical expressions of Multiple Endocrine Neoplasia type 1 (MEN1) syndrome or Hyperparathyroidism-Jaw Tumor (HPT-JT) syndrome, respectively.

Heterozygote inactivating mutations of the Calcium Sensing Receptor (*CaSR*) gene have been found in about 2% of FIPH cases [5]; 10 FIPH pedigrees with a loss-of-function *CaSR* mutation have been reported to date [6]. FIHP affected patients with *CaSR* mutations have elevated levels of PTH as consequence of development of PAs; no cases of PC have been reported. The *CaSR* gene encodes a G-protein-coupled transmembrane receptor, expressed prevalently in the parathyroids and the kidneys, able to “sense” the serum calcium concentration and control calcium homeostasis by regulating PTH secretion, and calcium reabsorption at renal tubular level. It is possible that specific inactivating mutations may have deleterious effect on parathyroid cell proliferation, even in the heterozygote state. However, the exact link between the calcium sensing activity of CaSR protein and the proliferative pathways remains to be elucidated.

Germline heterozygote activating mutations of the Glial Cells Missing Transcription Factor 2 (*GCM2*), encoding a transcription factor that acts as a master regulator of parathyroid development and may mediate the effect of calcium on PTH expression and secretion in parathyroid cells, were identified in FIHP probands by whole-exome sequencing, have been shown to co-segregate with the disease in affected members of the mutated FIHP families and confirmed in a prevalence-screening cohort of FIHP probands [7]. All the identified missense mutations were located within the amino acid 379–395 region of the protein, in the C-terminal conserved inhibitory domain (CCID), which is the only conserved region, apart from the N-terminal domain of GCM2. A mutated CCDI confers a gain of function and increases transcriptional activity to the GCM2 protein, suggesting that the *GCM2* gene acts as a proto-oncogene in the context of parathyroid tumorigenesis [7].

Warner et al. [8] linked a 1.7 Mb region on chromosome 2p13.3-14, located between D2S2368 and D2S358 microsatellite markers, to affected members of nine FIHP pedigrees. However, sequencing of the two most likely candidate genes in this region, *PPP3R1* and *PKR1*, failed to find any mutation and to identify a candidate gene for FIHP.

## 3. Inherited Syndromic Parathyroid Tumors

Inherited syndromic forms of parathyroid tumors include MEN1, Multiple Endocrine Neoplasia type 2A (MEN2A), Multiple Endocrine Neoplasia type 4 (MEN4), and HPT-JT syndrome.

### 3.1. Multiple Endocrine Neoplasia Type 1 (MEN1)

Parathyroid tumors occur in about 95% of MEN1 patients, being the first endocrinopathy in 90% of cases [9], manifesting as synchronous or asynchronous adenomas affecting all four glands during patient’s life. PC is extremely rare in MEN1 patients. Tumor development is caused by biallelic inactivation of the *MEN1* tumor suppressor gene, with the first gene copy being inactivated at birth by a heterozygote germinal mutation, inherited by the affected parent or, rarely, developed at the first stage of embryonic development, and the second wild type copy lost at parathyroid somatic level prevalently by loss of heterozygosity (LOH) at the 11q13 locus or, more rarely, by loss-of-function point mutations or small indels. Menin, the protein encoded by the *MEN1* gene, is an extremely pleiotropic tumor suppressor exerting various important anti-proliferative and anti-tumoral functions, such as regulation of gene expression, regulation of cell signaling, control of cell cycle progression, cell adhesion, cell mobility and apoptosis, maintenance of genome stability, and prevention/repair of DNA damage, via its direct interaction with numerous molecular partners [10]. The complete lack of functional domain homology with other known proteins makes it a challenge to elucidate the exact mechanisms leading to tumorigenesis of target neuroendocrine tissues in consequence of its loss, including parathyroids.

It has recently been shown that loss of menin in MEN1-related PAs is associated with an increased activity of the DNA (cytosine-5)-methyltransferase 1 (DNMT1), an enzyme responsible for methylation of cytosine residues of the CpG islands of DNA. Addition of methyl groups to CpG dinucleotides in gene promoters results in silencing gene expression. Hypermethylation of promoters of tumor suppressor genes, following menin loss, was demonstrated to be a common pro-oncogenic epigenetic change in MEN1 pancreatic neuroendocrine tumors [11], and a similar mechanism can be suspected also in *MEN1* loss-driven parathyroid tumorigenesis, both for the syndromic and the sporadic forms.

A direct autoregulatory network between miR-24-1, *MEN1* mRNA, and menin was demonstrated in parathyroid tissues of MEN1 patients [12]. MEN1 PAs still retaining a wild type copy of the *MEN1* gene presented increased levels of miR-24-1, associated with a complete silencing of the menin protein, suggesting a direct inhibitory action of miR-24-1 on MEN1 mRNA translation. Therefore, this epigenetic change may represent an intermediate, still reversible, step before the occurrence of the reversible genetic LOH of the *MEN1* gene, which inhibits menin expression and triggers MEN1 parathyroid tumorigenesis by mimicking the second inactivation hit on the *MEN1* gene expression [12].

A microarray profiled expression of 1890 human microRNAs (miRNAs) between MEN1 PAs with somatic *MEN1* LOH (*MEN1* LOH PAs) and MEN1 PAs still retaining one wild type copy of the *MEN1* gene (*MEN1* non-LOH PAs), showed 3 miRNAs as significantly differentially expressed between the two groups of adenomas [13]. miR-4258 resulted down-regulated in *MEN1* LOH PAs. This miRNA is predicted to target and inhibit the *CCDN1* gene, encoding the cyclin D1, a positive regulator of cell cycle and cell growth that has been found to be frequently over-expressed in parathyroid tumors [4]. It can be speculated that in the absence of wild type menin expression, the parathyroid cell loses the miR-4258-driven negative control of *CCND1* expression, leading to an increased expression of cyclin D1 and to an uncontrolled cell growth. miR-664 was up-regulated in *MEN1* non-LOH PAs. This miRNA is predicted to target the Cyclin Dependent Kinase Inhibitor 2C (*CDKN2C*) tumor suppressor gene, encoding the p18^/INK4c^, an inhibitor of the cyclin kinases CDK4 and CDK6, which negatively controls the cell cycle G1 progression, and whose somatic inactivating mutations have been found in sporadic PAs [14]. miR-1301 was up-regulated in *MEN1* LOH PAs with respect to both the *MEN1* non-LOH PAs and sporadic non-MEN1 adenomas. This miRNA is predicted to target various genes, previously demonstrated to have a role in the parathyroid pathophysiology: (1) the REarranged during Transfection (*RET*) proto-oncogene, whose germinal activating mutations are responsible for the multiple endocrine neoplasia type 2 (MEN2) syndrome; (2) the Cyclin Dependent Kinase Inhibitor 1B (*CDKN1B*) tumor suppressor gene, whose germinal inactivating mutations are responsible for MEN4 syndrome, and have also been found, at somatic level, in sporadic PAs [15]; (3) the *CCND2* gene, encoding the cyclin D2, a positive regulator of the G1 to S phase transition of the cell cycle and of cell proliferation; (4) the *CTNNB1*, gene encoding the β-catenin, a positive effector of the Wnt signaling positively regulating cell proliferation and involved in the regulation of cell adhesion; and (5) the *AP2S1* gene, whose germinal loss-of-function mutations have been associated with familial hypocalciuric hypercalcemia type 3, an inherited disorder characterized by a deregulation of PTH secretion and altered calcium homeostasis. These data suggest an important role of miRNA deregulation in MEN1 parathyroid tumorigenesis, which involves pro-oncogenic molecular pathways common to other syndromic and/or sporadic parathyroid tumors.

### 3.2. Multiple Endocrine Neoplasia Type 2A (MEN2A)

Parathyroid tumors develop only in the clinical subtype 2A of the MEN2 syndrome, affecting about 20% of MEN2A patients and manifesting as benign PAs in almost all cases, with single-gland or multiple-gland presentation, usually causing a mild PHPT. MEN2 syndrome is caused by germinal heterozygote activating mutation of the *RET* proto-oncogene, encoding the RET transmembrane tyrosine kinase receptor, which binds members of the glial cell line-derived neurotrophic factor (GDNF) family of extracellular signaling molecules. MEN2A subtype is caused by mutations in exons 10 and 11 of the gene, which specifically encodes the cysteine-rich extracellular domains of the RET protein, while mutations responsible for the MEN2B clinical subtype, which does not manifest any parathyroid tumorigenesis, are located in exons 15 and 16 and affect the tyrosine kinase intracellular domain of the RET protein. Therefore, genetic testing for the *RET* gene allows us to distinguish between the two clinical subtypes of the syndrome, to foresee clinical manifestations, and to exclude the risk of PAs in case of identification of a MEN2B-causing mutation. In particular, MEN2A is typically caused by a missense mutation replacing one of five extracellular cysteine residues in the juxtamembrane region of the RET receptor (Cys609, Cys611, Cys618, Cys620, and Cys634) in up to 95% of cases [1,16]. The substitution of one of the extracellular cysteine residues with another amino acid leaves the unpaired partner cysteine free to form intermolecular disulfide bonds with other adjacent receptor molecules, thereby generating covalent RET receptor dimers, being constitutively activated even in the absence of the natural ligand and possessing a high cell transforming activity [17]. The p.Cys634Arg substitution is found in 85% of MEN2A kindreds and possesses a higher transforming capacity [1]. Extremely rare non-cysteine mutations have been reported in MEN2A, such as Glu768, Leu790, Tyr791, Val804, and Ser891 in the tyrosine kinase domain, all having a lower transforming ability than those involving cysteines in the extracellular domain [18].

Currently, only two cases of metastatic PCs have been reported in MEN2A, one associated with the p.Cys634Tyr mutation [19] and one with the p.Cys618Arg rare missense variant in exon 10 of the *RET* gene [20].

### 3.3. Multiple Endocrine Neoplasia Type 4 (MEN4)

MEN4 is a clinical phenocopy of MEN1 caused by germinal heterozygote loss-of-function mutation of the *CDKN1B* gene, encoding the cyclin-dependent kinase inhibitor p27^Kip1^, which negatively regulates cell cycle progression and cell growth, primarily by binding and inhibiting the cyclin E/CDK2 complex [21]. Inactivating mutations of *CDKN1B* lead to reduced expression of p27^Kip1^, thereby resulting in an uncontrolled cell cycle progression. Dissimilarly from the *MEN1* gene, no somatic LOH at the 12p13 locus, containing the *CDKN1B* gene, was found in MEN4 PAs, suggesting this gene as a non-conventional tumor suppressor for which the down-regulation of p27^Kip1^ protein expression occurs at a post-transcriptional and/or post-translational level [22] by mechanisms that may regulate p27^Kip1^ stability, like as phosphorylation and ubiquitination [21]. The immunohistochemical staining of tumor tissues in MEN4 showed no expression of the p27^Kip1^ protein or a cytosolic mislocalization [21]. *CDKN1B* deletions lead to a truncated p27^Kip1^ protein rapidly degraded by the proteasome, while missense mutations lead to a reduced binding to interacting partners or a decreased nuclear localization [21]. Overall, *CDKN1B* mutations in MEN4 affect cellular localization and stability of the p27^Kip1^ or the ability to bind and inhibit CDK2 [23].

Parathyroid tumor-derived PHPT has been reported in up to 80–90% of patients with MEN4 [9], presenting at a later age and with a milder clinical phenotype compared to MEN1, and with a female predominance [24]. No cases of PCs have been described to date [25].

### 3.4. Hyperparathyroidism-Jaw Tumor Syndrome (HPT-JT)

HPT-JT syndrome is a rare inherited tumor syndrome characterized by development of parathyroid neoplasms (PAs and PCs) in over 90% of patients, and unusual bony lesions of the mandible and maxilla (ossifying fibromas). About 15% of HPT-JT patients manifest PC. A single gland is usually affected, but multiglandular disease may develop in more than 15% of cases [6].

HPT-JT has shown an autosomal dominant inheritance with incomplete age-dependent penetrance. Germinal inactivating mutations of the *CDC73* gene are detected in 50–75% of HPT-JT families [1,6]. Evidence of disease linkage to chromosome 1q31.2 was shown in some pedigrees negative for *CDC73* mutations [1]. *De novo CDC73* mutations have been identified in some cases, leading to mosaicism [1]. Hewitt et al. [26] found evidence of *CDC73* promoter methylation in a percentage of HPT-JT PCs, all without mutation of the *CDC73* gene, suggesting this epigenetic gene-silencing tool as another mechanism by which the *CDC73* gene inactivation may give rise to PCs. Almost all the *CDC73* mutations identified in HPT-JT are frameshift insertion/deletion variants (60%), creating a premature stop codon, and nonsense mutations (26%), resulting in a truncated inactive protein unable to reach the nucleus. Splicing site mutations (5%), or loss of initiator methionine mutations (3%), lead to an altered non-functioning protein or to the complete loss of protein translation, respectively. Missense mutations represent only 5% of HPT-JT cases [6]. HPT-JT patients bearing frameshift or nonsense mutations or large deletion of the *CDC73* gene are nearly 7-fold more likely to develop parathyroid cancer than patients harboring a missense mutation [27].

*CDC73* is a tumor suppressor gene that encodes a nuclear protein, named parafibromin, a component of the human PAF1 complex, which controls gene transcription by interacting with the subunit A of the RNA polymerase II, with the SUV39H1 histone methyltransferase complex (promoting H3K4 and H3K79 methylations), and with the RNF20/RNF40 ubiquitine ligase complex (promoting monoubiquitination of histone H2B at the lysine residue K120; H2BK120ub1). Moreover, parafibromin activates Wnt signaling by directly interacting with β-catenin and suppresses tumor growth via the down-regulation of cyclin D1 expression, inhibiting the G1 to S phase transition of the cell cycle and inducing apoptosis of tumor cell.

PAF1 complex interacts with the histone H1.2. Normally, H1.2 inhibits transcription of growth suppressive genes via modulation of chromatin structure [28]. In about half of parathyroid tumors bearing a *CDC73* mutation, and showing loss of parafibromin, a positive expression of H1.2 was found in more than 60% of cells, and the H1.2-regulated transcripts resulted as up-regulated (over 2-fold the mean expression values of normal parathyroid tissue) in 80% of *CDC73*-mutated parathyroid tumors, including HPT-JT PAs and PCs, FIHP parathyroid tumors and sporadic PCs [29], suggesting an increased H1.2-driven inhibition of cell growth suppressive genes and subsequent promotion of cell proliferation as a common pro-oncogenic mechanism in *CDC73*-mutated parathyroid cells.

*CDC73*-mutated parathyroid tumors are characterized by loss of H2BK120ub1, a histone modification that normally opens the chromatin structure permitting gene transcription and has a role in histone cross-talking and histone H3 methylation (H3K4me and H3K79me) [30]. Loss of H2BK120ub1 is an epigenetic change that has been shown in advanced cancers and, thus, may also be involved in *CDC73* loss-derived parathyroid tumorigenesis, both in HPT-JT-related and sporadic tumors.

Biallelic inactivation of *CDC73* is associated with loss of immunohistochemical staining of parafibromin in the nucleus. Loss of nuclear localization and activity of parafibromin leads to enhanced cell proliferation. Moreover, parafibromin-deficient tumors have shown a strong association with malignant behavior, with a younger age and larger tumor size, and present distinctive morphological (i.e., eosinophilic cytoplasm, very frequent presence of perinuclear cytoplasmic clearing, nuclear atypia and nuclear enlargement with distinctive coarse chromatin) and proliferative (extensive sheet-like growth rather than acinar architecture) features [31]. The same characteristic was found in sporadic PCs with somatic homozygote loss of wild type *CDC73* expression [31].

## 4. Sporadic Parathyroid Tumors

Sporadic parathyroid tumors occur as single-gland disease in almost all cases, usually by the age of 50 years, with a female/male ratio of about 3:1 [1]. The etiology of non-inherited parathyroid tumors remains largely unclear; older age, female gender and previous exposure to neck irradiation are considered main risk factors [4,32]. Somatic and, more rarely, germinal mutations in specific genes have been identified as responsible for the pathogenesis of sporadic parathyroid tumors in variable percentages of cases and with different genetic signatures distinguishing PAs from PCs.

Somatic inactivating mutations of the *MEN1* tumor suppressor gene have been demonstrated to be one of the main pathogenic events in non-inherited benign PAs, occurring in about 20–40% of cases [33]. LOH at the 11q13 locus was found in about 40% of sporadic PAs [1]. The common involvement of *MEN1* mutation/inactivation in sporadic PAs was also evidenced by two whole-exome studies. Newey et al. [34] showed the occurrence of somatic *MEN1* mutations in about 35% of analyzed PAs, in association with LOH at the 11q13 locus. Cromer et al. [35] found, through whole-exome profiling, the presence of a truncating mutation of the *MEN1* gene in 50% of analyzed PA cases, accompanied by LOH of the remaining wild type allele; the *MEN1* gene was found to also be mutated in 35% of the additional 185 PA samples analyzed by *MEN1* gene-targeting sequencing.

The over-expression of cyclin D1 protein is a common event in sporadic parathyroid tumors (20–40% of benign PAs and about 90% of malignant PCs) [4]. Cyclin D1 is a positive regulator of G1 to S phase transition of the cell cycle, through the activation of the cyclin-dependent kinases 4 and 6 (CDK4 and CDK6). The exact mechanisms responsible for cyclin D1 over-expression in parathyroid tumorigenesis are still largely unclear, but it is suspected that they involve copy number alterations or chromosomal rearrangements and trans-acting altered regulation of gene expression, rather than mutations of the *CCDN1* gene. A genome-profiling of sporadic PCs showed somatic amplification of the genomic region containing the *CCDN1* gene in 29% of analyzed cases, 80% of them mutually exclusive with cases harboring a *CDC73* somatic mutation [36]. Arnold et al. described a p15-q13 pericentromeric inversion in chromosome 11 in two non-related cases of benign PAs, which positioned the 5′-regulatory element of the *PTH* gene directly upstream to genes of the 11q13 locus, such as the *CCDN1* proto-oncogene, inducing over-expression of cyclin D1 protein [37]. A transgenic mouse model with the *Ccdn1* gene under the direct control of the regulatory element of the *Pth* gene, mimicking the chromosomal rearrangement found in human PAs, was used to study the role of cyclin D1 over-expression in parathyroid tumorigenesis. Mutated mice showed hyperplastic enlarged parathyroids with increased cell proliferation, enhanced PTH secretion and hypercalcemia, and developed chronic PHPT with characteristic abnormalities in bone [38], providing evidence of a direct role of cyclin D1 over-expression in uncontrolled parathyroid cell proliferation and in hypercellularity-derived excessive release of PTH. A further study on the same mouse model showed that the enhanced parathyroid cell proliferation was the early response to cyclin D1 over-expression, and temporarily preceded the dysregulation of the calcium-PTH axis [39].

Sporadic malignant PCs showed a strong association (70–100% of cases) with somatic *CDC73* mutations; LOH involving the *CDC73* locus on chromosome 1q31.2 has been reported in 50–55% of sporadic PCs [6]. The complete absence of nuclear staining for parafibromin is a histological hallmark of PCs, distinguishing these malignant tumors from PAs [40]. Two genome-profiling analyses also specifically focused on sporadic PCs confirmed the high rate of *CDC73* mutations associated with these malignant tumors. In the first study, Yu et al. [41] found *CDC73* inactivating mutations in 77.8% of the analyzed samples, associated with gene LOH (focal deletion or a whole chromosome locus loss) in 42.9% of the *CDC73* mutated cases. Two years later, Pandya et al. [36] showed the presence of a somatic *CDC73* mutation in 47% of the analyzed sporadic PC samples, 50% of them also bearing a germline inactivating variant of the gene, in the absence of a family history of PHPT and no clinical evidence of HPT-JT. Wei et al. [32] found two different somatic mutations of the *CDC73* gene in tissue samples from benign PAs, of which the p.Tyr54X nonsense mutation had been previously identified in malignant PCs [6], suggesting that sporadic PAs and PCs may share some common genetic features. Interestingly, germline *CDC73* mutations were also found in patients with apparently sporadic PCs and no known family history of PHPT or HPT-JT syndrome, suggesting the possibility of a HPT-JT incomplete form caused by an altered penetrance of the mutation or of a phenocopy [42]. As for HPT-JT-related PCs, the aberrant methylation of the *CDC73* promoter was also found in a similar percentage of sporadic PCs, but not in sporadic PAs [26].

Other rare somatic genetic defects have been found in tissue samples from sporadic parathyroid tumors. In two distinct studies on sporadic PAs, Costa-Guda et al. [14,15] found inactivating germline and somatic mutations in four cyclin-dependent kinase inhibitor genes (*CDKN1A*, *CDKN1B*, *CDKN2B* and *CDKN2C*), encoding, respectively, the p21^Cip1^, p27^Kip1^, p15^INK4b^ and p18^INK4C^ inhibitors of the cyclin-dependent kinases, and negative regulators of cell cycle progression and cell proliferation.

Three whole-exome sequencing studies performed on patients with sporadic PAs, both on tumor specimens and the corresponding blood-derived genomic DNA, confirmed the *MEN1* gene as the most common mutated/loss in sporadic PAs at the somatic level, and identified novel mutated genes that may represent low-frequency driver mutations in parathyroid tumorigenesis [32,34,35].

In a study by Newey et al. [34], only the *MEN1* gene resulted mutated in more than one of the analyzed sporadic PA samples; all the other somatically mutated genes were identified only in a single tumor sample and, thus, need to be further investigated to confirm a possible role in the development of parathyroid cancer. Interestingly, one of the analyzed PA samples, which showed a high mutation rate and LOH involving multiple chromosomes, had a somatic missense mutation (p.Gln290Arg) in the structural maintenance of the chromosome 3 (*SMC3*) gene and somatic point mutation (c.G546C) in the last base of the exon 8 of the protection of telomeres 1 (*POT1*) gene, resulting in the complete exon 8-skipping event. *POT1* encodes a single-stranded DNA-binding protein with a key role in maintaining telomere integrity and genome stability, and exon 8 encodes two domains that directly exert a telomere protection function [34]. Therefore, it is likely that the identified *POT1* and *SMC3* mutations may result in genome instability and contribute to the highly mutated genotype of this patient.

Cromer et al. [35] identified, by whole-exome screening, a missense mutation (p.Tyr641Asn; Y641N) of the Enhancer of Zeste 2 Polycomb Repressive Complex 2 Subunit (*EZH2*) gene in one PA sample, and confirmed the same mutation in an additional PA case by single gene-targeting sequencing. The same *EZH2* somatic mutation was identified in a PA sample from the Chinese population [32]. The *EZH2* gene encodes a protein that participates in the methylation of histone H3, in particular the H3K27 trimethylation (H3K27me3), which is a key epigenetic regulator of repression of gene expression, both during early development and in adult organisms, and thus is involved in the maintenance of the transcriptional repressive state of genes over successive cell generations. The identified mutation is a gain-of-function missense variant that has been demonstrated, by in vitro functional studies, to possess an increased H3K27me3 activity [43]. Increase of H3K27me3, associated with the biallelic inactivation of the *MEN1* gene, has been demonstrated in neuroendocrine tissue [44]. Cromer et al. [35] speculated that the Y641N activating mutation of *EZH2* could represent a phenocopy of *MEN1* loss-of-function mutations, in which the parathyroid tumorigenesis may be driven by common epigenetic changes, leading to the development of a PA phenotype. In addition, the *EZH2* gene amplification, with consequent over-expression of the EZH2 protein, contributing to the aberrant accumulation of active β-catenin in the nucleus and resulting in increased cell proliferation, is a common event in parathyroid hyperplasia, adenomas, and carcinomas, confirming a role of the enhanced expression of this gene as a pro-oncogenic factor in parathyroid cells [45].

In the Chinese population, Wei et al. [32] showed, in sporadic PAs, recurrent somatic mutations of the Additional Sex Combs Like 3 (*ASXL3*) gene, encoding a zinc finger domain-containing protein that plays a role in the regulation of gene transcription, which had never been reported in parathyroid cancer before.

The specific performance of whole-genome analysis on sporadic PCs confirmed the *CDC73* gene as the most commonly mutated, also in the sporadic form of these malignant tumors, and showed a distinctive set of mutated genes with respect to sporadic PAs [36,41]

Yu et al. [41] identified a germinal mutation associated with somatic LOH at the gene locus on chromosome 9, two somatic missense mutations and two somatic nonsense mutations of the Prune Homolog 2 with BCH Domain (*PRUNE2*) gene, that all failed to be found in 40 sporadic PAs, revealing mutations of this gene as a recurrent event in carcinoma and as a possible distinctive genetic signature of sporadic PCs. Among the functions of *PRUNE2* encoded protein there is the suppression of Ras homolog family member A activity, which results in suppression of oncogenic cellular transformation. Therefore, loss of function of this protein may drive malignant tumorigenesis of parathyroid cells.

Pandya et al. [36] found four recurrently mutated genes in sporadic PCs: *AKAP9*, *ZEB1*, *FAT3* and *ADCK1*, respectively, in 17.6%, 17.6%, 11.8% and 11.8% of analyzed cases. They found, in two unrelated patients with PCs, the recurrent p.Ile482Met mutation of the *ADCK1* gene, which encodes a putative kinase with unknown molecular function. *AKAP9* and *ZEB1* were found mutated in 3 cases, the first encoding a regulator of protein kinase A and presumably involved in multiple signal transduction pathways, and the second encoding a zinc finger transcription factor that acts as a transcriptional repressor and is shown to facilitate tumorigenesis and promote tumor metastasis [46,47]. Two different somatic truncating loss-of-function mutations of the *FAT3* gene were identified in two sporadic PCs, suggesting this gene as a novel candidate tumor suppressor gene in parathyroid carcinogenesis, possibly regulating the Wnt signaling. The same study showed that the PI3K/Akt/mTOR pathway is altered in 21% of PC cases. In particular, functionally established activating mutations of the *PIK3CA* gene, encoding the catalytic subunit of the PI3 kinase (PI3K), were found in three PCs, being mutually exclusive with *CDC73* mutations, thus indicating an independent oncogenic involvement of the PI3K/Akt/mTOR pathway in PCs. The PI3K/Akt/mTOR signaling plays a role in the regulation of cell growth and cell survival during stress; its deregulation as a consequence of *PIK3CA* mutations may alter the balance between cell proliferation and apoptosis, conferring a growth advantage to the mutated cell, and, presumably, also a metastatic competence [48].

In sporadic parathyroid tumors bearing *MEN1* or *CDC73* mutations, the deregulation of DNA methylation mechanism and/or of histone modifications, derived by menin or parafibromin loss, substantially concurs to the tumorigenesis, the same way it happens in inherited forms of MEN1- and HPT-JT-related parathyroid tumors.

As for other human malignancies, the deregulation of epigenetic mechanisms can cooperate with genetic alterations in parathyroid tumor development and growth, driving the tumor phenotype, and it is strongly suspected to be a main factor responsible for the wide heterogeneity in biological and clinical presentation of different neoplasms. However, as opposed to other human cancers that have shown a genome-wide DNA hypomethylation of the intragenic regions, parathyroid tumors do not appear to be affected by changes in global DNA methylation pattern compared to normal tissue [49,50]. On the contrary, the site-specific methylation of CpG dinucleotides, and the subsequent silencing of the regulated gene, in the promoters of tumor suppressor genes and genes known to be related to regulation of parathyroid pathophysiology, cell cycle progression and Wnt signaling appear to be a pro-oncogenic factor in parathyroid tumors. Promoter hypermethylation/silencing of the Adenomatous Polyposis Coli (*APC*) and Ras-association Domain Family Member 1A (*RASSF1A*) tumor suppressor genes have been demonstrated to be a common epigenetic change in parathyroid tumors [50,51,52].

The *RASSF1A* gene encodes a protein similar to the RAS effector proteins that is involved in induction of cell cycle arrest, thus *RASSF1A* silencing may result in uncontrolled cell growth. Juhlin et al. [51] found a *RASSF1A* promoter hypermethylation in 98% of the 55 analyzed PA samples, including three sporadic cases with somatic *CDC73* mutations, two sporadic cases with germinal *CDC73* mutations, and seven sporadic cases with somatic *MEN1* mutations. A further study by Sulaiman et al. [50] confirmed the frequent *RASSF1A* gene promoter hypermethylation in sporadic PAs (52%) and found this epigenetic change in all three analyzed PCs. Interestingly, the *RASSF1A* gene promoter was also shown to be hypermethylated in about 98% of MEN1 neuroendocrine tumors of the pancreas [11], suggesting the epigenetic inactivation of this gene as a common pathway of MEN1 loss-driven tumorigenesis in target endocrine tissues.

The *APC* gene encodes the homonymous protein APC, which is a strong negative regulator of the Wnt signaling by directly binding the β-catenin, promoting its rapid degradation and preventing its translocation to the nucleus. Hypermethylation of the *APC* promoter 1A was found by Juhlin et al. [51] in about 71% of analyzed sporadic PA samples. Sulaiman et al. [50] displayed a frequent hypermethylation of APC promoter 1A in PAs (56%), but not in PCs. The APC promoter 1A methylation was found to be an early pro-oncogenic event in colorectal tumorigenesis, and may also exert a similar effect in parathyroid cells.

The Secreted Frizzled-Related Protein 1 (*SFRP1*) gene encodes a potent antagonist of the Wnt signaling; *SFRP1* silencing by promoter hypermethylation constitutively activates the Wnt signaling pathway. Hypermethylation of the *SFRP1* gene promoter, as well as of other related family members, including *SFRP2* and *SFRP4,* has been reported in both benign and malignant parathyroid tumors in a genome-wide methylation analysis [52]. Conversely, Juhlin et al. [51] found hypermethylation of the *SFRP1* gene promoter only in PCs, and not in PAs.

Promoters of three additional tumor suppressor genes, *CDKN2B* and *CDKN2A*, encoding, respectively, the p15^INK4b^ and p16^INK4^ inhibitor of cyclin kinase, and, thus, negatively controlling cell cycle progression, and the Wilm’s tumor 1 (*WT1*) gene, encoding a transcription factor that plays an important role in cell growth and differentiation, have been found to be hypermethylated, and totally or partially silenced, in both benign and malignant parathyroid tumors [52].

Differentially hypermethylated promoters in PCs, with respect to normal parathyroid tissue, were found in genes involved in apoptosis regulation, such as *SOCS3* and *PYCARD*, or encoding transcription factors, such as *HOXC11*, *GATA4* and *HIC1*, with a significantly higher hypermethylation of *PYCARD*, *GATA4* and *HIC1* promoters in PCs compared to benign PAs [52,53].

Very recently, Singh et al. [54] showed that the expression of the Paired box 1 (*PAX1*) gene, encoding the homonymous transcription factor PAX1, which is normally active during parathyroid gland development, was significantly reduced in PAs by hypermethylation of the promoter and reduction of H3K9 acetylation at the chromatin *PAX1* promoter region, suggesting that this epigenetic-driven silencing of the *PAX1* gene possibly contributes to parathyroid tumorigenesis.

Among epigenetic changes, alterations in miRNA expression patterns, having pro-oncogenic effects, have been found in parathyroid hyperplasia, adenoma, and carcinoma, compared to normal parathyroid tissue [55,56]. In addition to having a role in tumorigenesis, distinct miRNAs signatures may help to distinguish different parathyroid tumor types.

The first study to profile differential expression of miRNAs in human PCs harboring a *CDC73* mutation and negative for parafibromin nuclear immunostaining, sporadic PAs, and normal parathyroid tissue was performed in 2010 by Corbetta et al. [55]. Despite the small sample size analyzed (4 PCs, 26 PAs and 2 normal parathyroids), the study evidenced specific miRNA signatures distinguishing among the three sample groups, and specifically characterizing PCs, with respect to both PAs and normal tissues. PCs were characterized by down-expressed miR-296 and up-regulated miR-503 and miR-222. miR-139 was similarly expressed between PCs and PAs, presenting a down-expression that characterized both the tumor types in comparison to healthy parathyroids. Immunostaining of putative targets of the three deregulated miRNAs showed, in PCs but not in PAs and normal tissue, a significant increased expression of hepatocyte growth factor-regulated tyrosine kinase substrate (HGS), a protein that has been found to be highly expressed in various human malignancies and is a direct target of miR-296. In the HeLa cells, over-expression of HGS has been associated with increased β-catenin and Wnt signaling, and loss of E-cadherin [57], which promote, respectively, cell proliferation via activation of cyclin D1, and cell invasion and metastases. Since the Wnt pathway has often been found to be deregulated in parathyroid tumors, similar HGS-mediated pro-oncogenic and pro-metastatic mechanisms and a role of miR-296 as tumor suppressor may also be hypothesized in PCs. The *CDKN1B* gene is a demonstrated target of miR-222 [58]. Nuclear immunostaining of the encoded p27^Kip1^ protein showed a decreased expression in PAs and an almost complete loss in PCs, suggesting the up-regulation of miR-222 accompanied by absence of nuclear immunoreactivity of p27^Kip1^ as a molecular marker of PCs.

Interestingly, the subset of over-expressed miRNAs in PCs belongs to the largest human primate-specific miRNA cluster, spanning a 100 kb long region on chromosome 19q13.4 (C19MC) and accounting for about 8% of all known human miRNA genes. C19MC and the adjacent smaller miR-371-3 cluster on 19q13.4 locus have been shown to be involved in stem cell biology and human tumorigenesis, and found to be activated by specific chromosomal rearrangements in thyroid adenomas [59]. Vaira et al. [60] analyzed the expression of 15 and 3 miRNAs belonging, respectively, to the C19MC and miR-371-3 clusters in a series of 15 PCs, 5 matched metastases, 24 PAs, and 6 normal parathyroids. Expression of miRNAs did not show any difference between benign PAs and healthy tissues. Conversely, PCs and metastatic lesions showed a characterizing miRNA expression profile, distinctive from PAs. In particular, the over-expression of miR-517c, located within the C19MC cluster, was shown to be associated with PCs and matched metastases, and presented the most significant expression difference between carcinomatous samples and both PAs and normal parathyroids, confirming the deregulated expression of miRNAs of the C19MC cluster as a possible marker and/or regulator of parathyroid malignant carcinogenesis and tissue invasion. Copy number variations at 19q13.4 locus were significantly associated with miR-517c over-expression, suggesting that the up-regulation of this miRNA in PCs could be the consequence of this mechanism. Hypomethylation of the C19MC promoter was shown in 100% of metastasis samples, 58% of PCs, and only 29% of PAs. Interestingly, miR-517c expression levels positively correlated with serum calcium and PTH values and with tumor weight, supporting a role of this miRNA in determining malignant clinical features of the tumors [60].

A further study [56] performed on 9 PCs, 12 PAs, 15 hyperplastic parathyroids and 4 normal parathyroids identified, after miRNA microarray profiling with the application of a false discovery rate <0.01, a set of 24 miRNAs as differentially expressed between PAs and PCs. Validation analysis identified three miRNAs as significantly down-regulated in PCs compared with PAs (mirR-126-5p, miR-26b and miR-30b), with the miR-126-5p appearing to be the most accurate molecular differentiator between the two types of tumors.

Very recently, some studies have investigated the contribution of long non-coding RNAs (lncRNAs) to the development of benign and malignant parathyroid tumors.

The first study [61] indicated the presence of a distinctive expression pattern of two specific lncRNAs between PCs and PAs, with the over-expression of the lncRNA PVT1 and the down-regulation of the lncRNA GLIS2-AS1 in PCs, with respect to PAs, being two distinctive molecular markers of malignant tumors. The lncRNA PVT1 was previously demonstrated to promote tumor cell proliferation in lung cancer by recruiting the EZH2 transcriptional repression factor, a protein demonstrated to have a role in parathyroid carcinogenesis, to the large tumor suppressor kinase 2 (LATS2), repressing its transcription [62]. No studies on cancer are available, to date, on the lncRNA GLIS2-AS1.

A tissue microarray analysis of lncRNA expression patterns, associated with immunohistochemical evaluation of tumor features in normal, hyperplastic, and benign and malignant neoplastic parathyroid samples, found the expression of lncRNAs ROR, HOTAIR and MALAT1 in all four of the parathyroid tissues analyzed, including both healthy and tumoral glands [63]. Only the lncRNA ROR showed a decreasing expression during the tumor progression from PAs to PCs, suggesting that this lncRNA may act as a tumor suppressor in parathyroid tumors.

A third study [64] analyzed the expression of known human lncRNAs in two series of non-familial PAs and PCs, and in healthy parathyroids as control, finding that up-regulation of lncRNA BC200 specifically characterized PCs and discriminated these malignant tumors from control tissues, PAs, and aPAs. Two lncRNAs, lncRNA-VLDLR and ZFAS1, were both significantly over-expressed in PAs and PCs with respect to normal parathyroids. Mutations of the *MEN1* gene were shown to influence the expression of 6 lncRNAs (NEAT1, BC200, HOXA3as, SNHG6, HAR1B, and ZFAS1), all presenting an increased expression in PAs with the *MEN1* gene loss compared to PAs without *MEN1* mutations. Similarly, PCs bearing a *CDC73* mutation showed a significantly up-regulated BC200 expression compared to that of PCs with wild type parafibromin, in association with malignant clinical features, such as higher circulating levels of PTH and calcium ion.

## 5. Conclusions

Parathyroid tumors show distinct cellular, histological, and molecular heterogeneity, which makes it difficult to pre-operatively distinguish PAs, aPAs, and PCs. Only extremely rare cases of PCs have been associated with mutations of the *MEN1* tumor suppressor gene and the *RET* proto-oncogene. To date, no cases of malignant PCs have been associated to mutations in the *CDKN1B* or other genes encoding for cyclin dependent kinase inhibitors.

Inactivation of the *CDC73* tumor suppressor gene is a genetic marker of risk for development of PCs, both in the context of HPT-JT syndrome (about 15% of cases) and as sporadic cancer. *CDC73* inactivation-related loss of parafibromin activity and immunostaining appears to be a biomarker of PC. Different genetic and epigenetic signatures have been associated with PAs or PCs. Main genetic and epigenetic signatures characterizing parathyroid carcinomas, and distinguishing these malignant tumors from benign PAs, are reported in Table 2.

The evaluation of miRNA expression deregulation appears to be a promising biomarker to distinguish different types of parathyroid tumors, and miRNA signatures specifically characterizing PCs have been identified. miRNA deregulation may confer a growth advantage and a metastatic potential to tumor cells in PCs compared to both benign parathyroid tumors and healthy glands. Down-regulation of miR-296 [55] and miR-126-5p [56] and up-regulation of miR-517c [60] appear to be the best molecular discriminators between PCs and PAs. The global score based on the miR-296, miR-222, and miR-503 gene-set expression has been shown to discriminate both normal and adenomatous parathyroid samples from PC [55]. The expression of specific lncRNAs has also been shown to be a promising molecular signature able to distinguish different human parathyroid tumor histotypes. However, the contribution of lncRNAs to the development of benign and malignant parathyroid tumors and their possible correlation with genetic alterations causing the disease must still be elucidated. Further studies are needed to confirm and validate these preliminary findings on independent and larger numbers of parathyroid tumor samples, and to associate the molecular signatures more specifically with clinical phenotypes and prognosis.

## Figures and Tables

**Table 1:** Summary of main genetic, epigenetic and molecular signatures associated with different types of sporadic and inherited parathyroid tumors.

Disease	Mean Age of Onset	Tumor Presentation	Genetic Signature(s)	Molecular Features	Epigenetic Signatures
**1. Sporadic parathyroid tumors**					
Sporadic isolated parathyroid adenoma	Commonly in the sixth decade of life.	Single-gland adenoma.	Somatic biallelic inactivation of the *MEN1* tumor suppressor gene in 20–40% of cases. p15-q13 pericentromeric inversion in chromosome 11 in about 5% of cases. Inactivating mutation of the *CDC73* tumor suppressor gene in 2–4% of cases. Rare somatic and germinal inactivating mutations of the *CDKN1A*, *CDKN1B*, *CDKN2B* and *CDKN2C* genes. Activating p.Tyr641Asp missense mutation of the *EZH2* gene in few cases.	Deregulation/loss of expression of menin protein in 20–40% of cases. Over-expression of the cyclin D1 protein in 30–40% of cases. Down-regulation of parafibromin expression in less than 5% of cases.	Hypermethylation (and silencing) of promoters of *RASSF1A*, *APC*, *SFRP1*, *SFRP2, SFRP4, CDKN2B*, *CDKN2A*, *WNT1* and *PAX1* genes.
Sporadic isolated parathyroid carcinoma	Commonly in the fifth decade of life.	Single-gland carcinoma.	Somatic biallelic inactivating mutations/loss of the *CDC73* tumor suppressor gene in 70–100% of cases. Amplification of the genomic region containing the *CCDN1* gene in about 30% of cases. Somatic and germinal inactivating mutations of the *PRUNE2* gene.	Deregulation/loss of expression of parafibromin protein in 70–100% of cases. Complete absence of nuclear staining for parafibromin. Over-expression of the cyclin D1 protein in about 90% of cases.	Hypermethylation (and silencing) of promoters of *RASSF1A*, *SFRP1*, *SFRP2, SFRP4, CDKN2B*, *CDKN2A*, *WNT1*, *SOCS3*, *PYCARD*, *HOXC11*, *GATA4* and *HIC1* genes. Down-regulation of miR-296, miR-126-5p, miR-26b and miR-30b. Up-regulation of miR-222, miR-503 and miR-517c. Down-regulation of lncRNA GLIS2-AS1. Up-regulation of lncRNA PVT1 and lncRNA BC200.
**2. Inherited isolated parathyroid tumors**					
Familial isolated hyperparathyroidism (FIHP)	Variable, but usually about two decades before the sporadic form of parathyroid cancer.	Multiple-gland tumors.	The specific genetic cause of FIHP has not yet been clearly identified. Inactivating mutations of the *MEN1* and the *CDC73* tumors suppressor genes and of the *CaSR* gene, as well as activating mutations of the *GCM2* gene, have been reported in some cases.	Loss of menin and parafibromin has been seen in a percentage of FIHP pedigrees.	Not reported.
**3. Inherited syndromic parathyroid tumors**					
Multiple Endocrine Neoplasia Type 1 (MEN1)	During the third decade of life.	Multiple-gland adenomas (all the four parathyroids are affected during life). Extremely rare cases of aPAs and PCs.	Germinal heterozygote inactivating mutation, associated with somatic inactivation/loss of the second copy of the *MEN1* tumor suppressor gene, mainly by LOH, or, rarely, by intragenic mutations.	Loss of wild type menin expression. Loss of nuclear localization of the menin protein.	Increased activity of DNMT1. Increased expression of miR-24-1 in PAs without *MEN1* LOH.
Multiple Endocrine Neoplasia Type 2A (MEN2A)	During the fourth decade of life.	Single-gland or multiple-gland adenomas (1 to 4 glands can be affected during life). Only two cases of metastatic PCs have been reported.	Germinal heterozygote dominant activating mutations in exons 10 and 11 of the *RET* proto-oncogene (p.Cys634Arg missense mutation in 85% of cases).	Homodimerization of the RET receptor in absence of ligand. Constitutively active RET-mediated signal transduction.	Not reported.
Multiple Endocrine Neoplasia Type 4 (MEN4)	During the fourth decade of life.	Multiple-gland adenomas (all the four parathyroids are affected during life). No cases of aPAs and PCs have been reported.	Germinal heterozygote loss-of-function mutations of the *CDKN1B* tumor suppressor gene.	Reduced/absent nuclear expression of the p27^Kip1^ cell cycle inhibitor protein.	Not reported.
Hyperparathyroidism-Jaw Tumor syndrome (HPT-JT)	Between the third and fourth decades of life.	Single- or multiple-gland adenomas in about 85% of cases; malignant carcinomas in up to 15% of cases.	Germinal heterozygote inactivating mutation with somatic biallelic inactivation/loss of the *CDC73* tumor suppressor gene.	Loss of parafibromin expression. Complete absence of nuclear staining for parafibromin.	Positive expression of the histone H1.2. Loss of the H2BK120ub1 histone modification.

aPAs = atypical parathyroid adenomas; PCs = parathyroid carcinomas; LOH = loss of heterozygosity; DNMT1 = DNA (cytosine-5)-methyltransferase 1; PAs = parathyroid adenomas.

**Table 2:** Genetic and epigenetic signatures characterizing parathyroid carcinomas, with respect to benign adenomas.

**Mutated Genes in Parathyroid Carcinomas**						
**Gene**	**Encoded Protein**	**Gene Function**	**Genetic Variations in PCs**	**Frequency in PCs**	**Molecular and Cellular Effects of Gene Variations**	**Molecular Signatures in PC Cells**
*CDC73*	Parafibromin	Tumor suppressor gene involved in the regulation of gene transcription, mRNA elongation and processing, and cell cycle progression.	Biallelic inactivating mutations/gene loss (germinal and/or somatic)	Mutations in 70–100% sporadic PCs [6] 1q31.2 LOH in 50–55% of sporadic PCs [6] Mutations 50–75% of HPT-JT families [1,6]	Altered elongation and processing of gene transcripts. Loss of nuclear transduction of the Wnt signaling. Increased expression of cyclin D1 and enhanced cell proliferation.	Negative immunostaining for parafibromin.
*PRUNE*	Prune Homolog 2 with BCH Domain (PRUNE2)	Tumor suppressor gene involved in the suppression of Ras homolog family member A activity, which results in inhibition of oncogenic cellular transformation.	Biallelic inactivating mutations/gene loss (germinal and/or somatic)	18.2% (4/22) of sporadic PCs [41]	The biallelic inactivation suggests loss of tumor suppressor activity and subsequent loss of control over cellular transformation.	Still unknown.
*AKAP9*	A-Kinase Anchoring Protein 9 (AKAP9)	Encoding a member of the A-kinase anchor proteins that regulates cellular localization and functions of the protein kinase A.	Biallelic inactivating mutations (somatic)	17.6% (3/17) of sporadic PCs [36]	The biallelic inactivation suggests loss of a putative tumor suppressor activity and subsequent loss of the correct cellular localization and function of the protein kinase A.	Still unknown.
*ZEB1*	Zinc Finger E-Box Binding Homeobox 1 (ZEB1)	Proto-oncogene encoding a zinc finger transcription factor that acts as a transcriptional repressor, represses E-cadherin promoter and induces the epithelial-mesenchymal transition (EMT).	Heterozygote somatic mutations	17.6% (3/17) of sporadic PCs [36]	The effects of identified mutations have not been evaluated yet, but activating mutations are suspected to promote EMT and tumor invasion and metastases.	Still unknown.
*ADCK1*	AarF Domain Containing Kinase 1 (ADCK1)	Encoding a putative kinase protein whose function are still completely unknown.	A recurrent heterozygote somatic missense mutation	11.8% (2/17) of sporadic PCs [36]	Function of the ADCK1 protein and effects of the identified missense mutation are still completely unknown.	Still unknown.
*FAT3*	FAT Atypical Cadherin 3 (FAT3)	Suspected tumor suppressor gene, presumably involved in the regulation of Wnt signaling. The exact biological functions have not been elucidated yet.	Biallelic truncating mutations (somatic)	11.8% (2/17) of sporadic PCs [36]	The effects of identified inactivating mutations have not been evaluated yet.	Still unknown.
**Hypermethylated Gene Promoters in Parathyroid Carcinomas**						
**Gene**	**Encoded Protein**	**Gene Function**	**Frequency in PCs**		**Molecular and Cellular Effects of Gene Promoter Methylation**	
*HIC1*	HIC ZBTB Transcriptional Repressor 1 (HIC1)	Encoding a transcription repressor that inhibits expression of the E2 transcription factor 1 (E2F1), by directly binding its promoter, and positively modulates p53 function by repressing transcription of the deacetylase SIRT1 that can inactivate p53 by deacetylation.	100% (5/5) of sporadic PCs [53]		No specific functional studies have been performed about the effect of transcription repression of the *HIC* gene in parathyroid tumors. Silencing of the *HIC* gene may result in reduction/loss of p53 tumor suppressor activity.	
*PYCARD*	PYD and CARD domain containing protein (PYCARD)	Encoding an adaptor protein, composed by a PYD domain and a CARD domain that promotes caspase-mediated apoptosis.	Not reported.		No specific functional studies have been performed about the effect of transcription repression of the *PYCARD* gene in parathyroid tumors. Silencing of the *PYCARD* gene may result in reducing apoptosis of cancer cells.	
*GATA4*	GATA Binding Protein 4 (GATA4)	Encoding a member of the GATA family of zinc-finger transcription factors that is involved in gene transcription regulation.	Not reported.		No specific functional studies have been performed about the effect of transcription repression of the *GATA4* gene in parathyroid tumors. *GATA4* down-regulation, by promoter hypermethylation, has been reported in various human malignancies.	
**Deregulated miRNAs in Parathyroid Carcinomas**						
**miRNA**	**Known Target mRNA(s)**	**Biological Function of Targeted mRNA(s)**	**Variation in PCs**		**Effects of miRNA Expression Deregulation**	
miR-296	*HGS*	HGS protein sorts monoubiquitinated membrane proteins into the multivesicular body, targeting these proteins for lysosome-dependent degradation.	Down-regulated		Down-regulation of miR-296 resulted in over-expression of HGS protein in PC samples [55]. In Hela cells the over-expression of HGS has been associated with increased β-catenin and Wnt signaling, and loss of E-cadherin, which promote, respectively, cell proliferation, and cell invasion and metastases [57].	
miR-126-5p	*EGFL7*	*EGFL7* gene encodes the epidermal growth factor-like domain 7 (EGFL7) protein that is involved in cellular migration and angiogenesis.	Down-regulated		miR-126-5p expression has been shown to repress cell proliferation and inhibit metastasis development in some human malignancies. Down-regulation of miR-126-5p results in over-expression of EGFL7 protein, presumably favoring tumor growth, neo-angiogenesis and leading to an increased cell migration.	
miR-26b	*PTEN*	The *PTEN* gene is a tumor suppressor gene encoding a phosphatidylinositol-3,4,5-trisphosphate 3-phosphatase protein (PTEN), which negatively regulates the intracellular levels of phosphatidylinositol-3,4,5-trisphosphate and the AKT/PKB signaling pathway.	Down-regulated		No specific functional studies have been performed about the effect of miR-26b down-regulation in parathyroid tumors. miR-26b overexpression showed to enhance survival, proliferation and migration of endothelial cells via inhibiting *PTEN* expression [65].	
miR-30b	*TRIM27*	The *TRIM27* gene encodes a member of the tripartite motif family (TRIM27) that interacts with the enhancer of polycomb protein and represses gene transcription.	Down-regulated		No specific functional studies have been performed about the effect of miR-30b down-regulation in parathyroid tumors. miR-30b has been shown to target the TRIM27-PI3K/Akt axis, and miR-30b over-expression showed to significantly repress cell viability, proliferation, migration, and invasion in hepatocellular carcinoma cells [66].	
miR-222	*CDKN1B*	The *CDKN1B*-encoded p27^Kip1^ protein is a cyclin-dependent kinase inhibitor, which negatively regulates cell cycle progression and cell growth.	Up-regulated		Over-expression of miR-222 resulted in almost all the complete loss of p27^Kip1^ expression and nuclear localization in PC samples [55].	
miR-503	*CCDN1*	The *CCDN1* gene encodes the cyclin D1, a positive regulator of cell cycle progression by promoting the G1 to S phase transition through activation of CDK4 and CDK6.	Up-regulated		Cyclin D1 displayed a heterogeneous immunoreactivity in PC samples with up-regulated miR-503 [55].	
miR-517c	Not reported	n.a.	Up-regulated		miR-517c expression levels were correlated with serum calcium and PTH levels, and higher expression levels of miR-517c were positively correlated with increased tumor weight [60].	
**Deregulated lncRNAs in Parathyroid Carcinomas**						
**lncRNA**	**Known lncRNA Biological Function(s)**		**Variation in PCs**		**Effects of lncRNA Expression Deregulation**	
lncRNA GLIS2-AS1	Still unknown.		Down-regulated		No studies on cancer are available, to date, on the lncRNA GLIS2-AS1.	
lncRNA PVT1	The *PVT1* gene has been identified as a candidate oncogene. Increased copy number and overexpression of this gene have been associated with many types of human cancers.		Up-regulated		No specific functional studies have been performed about the effect of lncRNA PVT1 up-regulation in parathyroid tumors. Some studies showed that lncRNA PVT1 promotes cancer cell proliferation partly by binding to *EZH2,* a transcriptional repressor that demonstrated to act as pro-oncogenic factor in parathyroid carcinogenesis [62].	
lncRNA BC200	lncRNA BC200 is a protein-interacting non-coding RNA presumably involved in the regulation of translation repression.		Up-regulated		lncRNA BC200 expression has been found to be substantially increased in certain human tumors, and a direct role in cell migration, proliferation and survival has been proposed [67].	

PCs = Parathyroid Carcinomas; miRNAs = microRNAs; HGS = Hepatocyte Growth Factor-Regulated Tyrosine Kinase Substrate; lncRNAs = Long Non-coding RNAs; n.a. = Non-applicable.

## Data Availability

Non applicable to this review.
